# From Erythromycin to Azithromycin and New Potential Ribosome-Binding Antimicrobials

**DOI:** 10.3390/antibiotics5030029

**Published:** 2016-09-01

**Authors:** Dubravko Jelić, Roberto Antolović

**Affiliations:** 1 Fidelta Ltd., Prilaz baruna Filipovića 29, HR-10000 Zagreb, Croatia; dubravko.jelic@gmail.com; 2 Department of Biotechnology, University of Rijeka, Radmile Matejčić 2, HR-51000 Rijeka, Croatia

**Keywords:** macrocycles, macrolides, quinolones, ribosome binding, dual-binding inhibition, azithromycin, erythromycin

## Abstract

Macrolides, as a class of natural or semisynthetic products, express their antibacterial activity primarily by reversible binding to the bacterial 50S ribosomal subunits and by blocking nascent proteins’ progression through their exit tunnel in bacterial protein biosynthesis. Generally considered to be bacteriostatic, they may also be bactericidal at higher doses. The discovery of azithromycin from the class of macrolides, as one of the most important new drugs of the 20th century, is presented as an example of a rational medicinal chemistry approach to drug design, applying classical structure-activity relationship that will illustrate an impressive drug discovery success story. However, the microorganisms have developed several mechanisms to acquire resistance to antibiotics, including macrolide antibiotics. The primary mechanism for acquiring bacterial resistance to macrolides is a mutation of one or more nucleotides from the binding site. Although azithromycin is reported to show different, two-step process of the inhibition of ribosome function of some species, more detailed elaboration of that specific mode of action is needed. New macrocyclic derivatives, which could be more potent and less prone to escape bacterial resistance mechanisms, are also continuously evaluated. A novel class of antibiotic compounds—macrolones, which are derived from macrolides and comprise macrocyclic moiety, linker, and either free or esterified quinolone 3-carboxylic group, show excellent antibacterial potency towards key erythromycin-resistant Gram-positive and Gram-negative bacterial strains, with possibly decreased potential of bacterial resistance to macrolides.

## 1. Introduction

It is well known that macrocyclic compounds have great potential for broad use in the treatment of different diseases and therefore make very interesting molecules. Most of macrocyclic drugs are predominantly used for the treatment of infectious diseases, but they have also been used in the treatment of cancer, auto-immune, and inflammatory diseases [1,2,3,4,5,6,7,8], since they possess significant anti-inflammatory and immunomodulatory properties [9,10,11,12,13], as well as tuberculostatic, antifungal, antiparasitic, antimalarial, antiviral, and antitumor properties [5,6,7,14,15,16,17]. The best known macrocyclic structures are macrolides, natural compounds produced by *Streptomyces* species that are the most commonly used class of antibiotics, and newly-synthesized macrocycles that also belong to the macrolide or cyclic peptide class. The first 14-membered macrolide, erythromycin A, has been in clinical use since 1952. Erythromycin is active against Gram-positive and some Gram-negative microorganisms and is used in treatment of respiratory, gastrointestinal, and genital tract infections, as well as skin and soft tissue infections [18]. To improve acidic stability and oral bioavailability of erythromycin A, the first generation of natural or semisynthetic macrolides such as spiramycin [19], roxithromycin [20], dirithromycin [21], and clarithromycin [22] were prepared and introduced to medical practice. Discovery of the first 15-membered macrolide—azithromycin, characterized by a basic nitrogen atom inserted into the macrocyclic ring, represented a breakthrough in the macrolide antibiotic era. Azithromycin became one of the best-selling branded antibiotics worldwide.

Structural and biochemical binding information is now available on ribosome-targeting antibiotics in various species, providing insight into principles of targeting and macrolide binding [23,24]. Macrolides, as a class of compounds, express their antibacterial activity by either blocking nascent proteins progression through their exit tunnel, or by paralyzing peptide bond formation at the peptidyl transferase center [23]. Only small macrolides, such as the 12-member macrolactone ring, bind to the peptidyl transferase center. The secondary structure of 23S rRNA is folded due to base pairing and forms six domains, numbered I to VI. The tertiary structure of the rRNA is held together primarily by long-distance RNA-RNA interactions and by proteins [25]. Chemical modifications of the macrolides have a direct influence on the differences in their binding modes as well as the resistances towards the antibiotics. This insight is of fundamental importance for the design of more potent macrolides that could overcome bacterial resistance [26].

A novel class of macrolide antibiotics, named “macrolones”, have been derived from azithromycin, and comprising macrocyclic moiety, linker and either free or esterified quinolone 3-carboxylic group [27,28]. They show excellent antibacterial potency towards key erythromycin-resistant Gram-positive and Gram-negative bacterial strains. Compared to azithromycin, most of the new compounds exhibit improved in vitro potency against the key respiratory pathogens [27,28,29]. These findings create new opportunities for in silico modeling and in vitro optimization work to produce more potent and more selective compounds, which would be less prone to bacterial resistance.

## 2. Macrolides and Their Mode of Action as Anti-Infectives

The era of modern anti-infective drug discovery started in 1928 when Alexander Fleming discovered (by chance) the first antibiotic from mold: penicillin from *Penicillium notatum*. Together with the discovery of the cephalosporins (from *Cephalosporium acremonium*), the penicillins are part of a large group of beta-lactams, the first generation of antibiotics. Another significant group of antibiotics were the tetracyclines, developed initially from a product of *Streptomyces aureofaciens* (chlorotetracycline). Oxytetracycline, a product of *Streptomyces rimosus*, was discovered in 1950. The macrolides represent a third family of well-known oral antibiotics. Medically-important macrolide antibiotics were originally characterized by a 12-membered (methymycin-like), 14-membered (erythromycin-like), or 16-membered (josamycin-like) lactone ring to which amino and neutral side-sugars are attached A.

**Figure 1:**
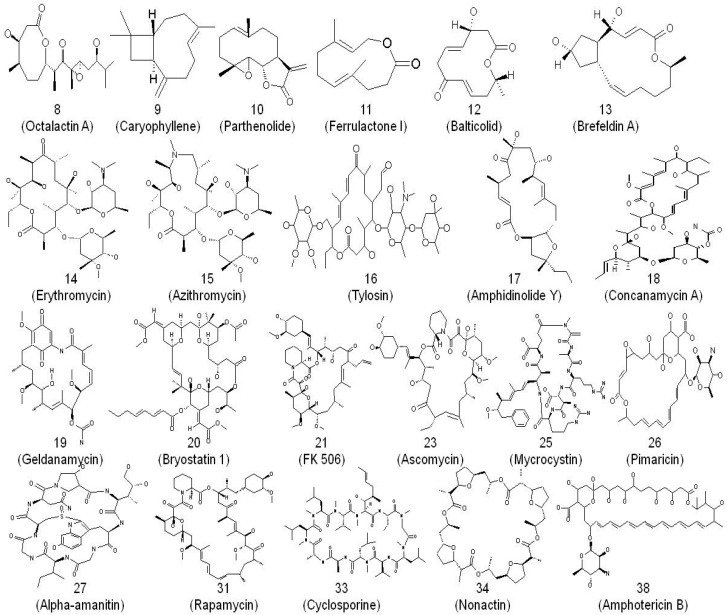
Examples of macrolide diversity [31], where macrocyclic ring lactones vary in size from eight-membered up to 62-membered rings. Numbers indicate the size of the ring.

Originally applied to compounds originally extracted from natural sources, macrolides, as a broader chemical term, now encompasses all macrocyclic ring lactones varying in size from eight-membered up to 62-membered rings (Figure 1).

Actinomycetes are very effective in production of bioactive compounds, such as macrolides (antibiotics), rapamycin (immunosuppressant) [13], avermectin (antiparastic) [15], nystatin (antifungal) [14], and especially with respect to antitumor compounds (doxorubicin, bleomycin) [16,17]. The importance and use of macrocyclic compounds in immunosuppression, inflammation, cancer, and infection are rapidly growing. The chemical modifications of existing macrocyclic drugs are in progress and their new therapeutic characteristic have improved further. Inflammatory cells bioaccumulate macrolides and transport them to the infected tissues. During the treatment of bacterial infections, macrolide accumulation in inflammatory cells plays an important role, since inflamed tissue releases a whole range of chemoattractant molecules, and polymorphonuclear cells, which are loaded with the antibacterial agent are, therefore, concentrated in the inflamed tissue. Bacterial components activate and degranulate inflammatory cells, and the macrolide is released into the surrounding tissue, contributing to faster clearance of the infectious pathogen [30].

The medically most important macrolide antibiotics are those structured from 12- to 16-membered large lactone rings with one or more sugar moieties, generally desosamine and cladinose, linked to the macrocyclic core [32]. Macrolides express their antibacterial activity by binding themselves to the bacterial 50S ribosomal subunits and inhibiting protein synthesis. More specifically, they have interactions with the region of the structure of the 50S subunit defining the catalytic core and the ribosomal exit tunnel (peptidyl transferase-associated region) [26]. They bind to the 23S rRNA at the nascent peptide exit tunnel and inhibit the growth of nascent peptides [33].

The ribosome interacts with different molecules in the process of translation, and the global and local structures of ribosome complexes are continuously changed to perform different functions during different stages. The subunit rotations induce conformational change that occurs during elongation, forming a rotated structure [34]. The first high-resolution X-ray crystal structures of a eukaryotic ribosomes was resolved almost two decades ago [35,36], showing ribosomes as interesting biological targets for their complexity and importance in the functioning of the whole cellular machinery. Antibiotics, interfering with ribosomal dynamics and mobility, can facilitate miscoding and influence the protein translation rate, while the degree of attenuation depends on the structure and binding position of antibiotics into the ribosome [37]. The strength of translation attenuation depends on the pause of ribosome function and the degree of ribosome stalling. All the stalling factors induce global conformational changes [38].

The active site of the ribosome is the peptidyl-transferase center in which the peptide bond among amino acids of the newly synthesized protein is formed [39]. Crystal structure of the 50S subunit clearly shows that the environment of the peptidyl transferase center is made of the 23S RNA. The many classes of ribosomal antibiotics (natural or synthesized chemical compounds by its origin) target the peptidyl transferase center [37]. Several antibiotics including the macrolides bind to 23S rRNA close to the pocket in the nascent peptide exit tunnel, approximately 8–10 Å away from the peptidyl transferase center of the 50S subunit. The consequence of that is the bacteriostatic or bactericidal effect of that class of antibiotics [40]. Due to binding closely to the peptidyl transferase center, they effectively block the formation of the peptide bond and extension of the peptide chain leading to dissociation of peptidyl-tRNA [41]. Amino acid and nucleotide identities in the binding pocket determine the binding of antibiotics since mutations in the binding pocket make the bacteria resistant to antibiotics [33]. Macrolide antibiotics, which include the representative drug erythromycin, bind in the exit tunnel near the peptidyl transferase center, contacting RNA (A2058 and A2059) and protein (L4, L22). Variability in macrolide structures has influence on the binding and inhibitory modes. Therefore, extensive efforts in drug discovery and design of new and improved antibacterial macrocyclic agents continue in order to discover new compounds with better profile toward resistant pathogens.

## 3. From Erythromycin to Azithromycin

The first 14-membered macrolide, erythromycin A, isolated from the actinomycete *Streptomyces erythreus* (*Saccharopolyspora erythraea*), has been in human use since 1952. Erythromycin has an antimicrobial spectrum similar to that of penicillin, and was widely used for patients who are allergic to penicillin. It exerts bacteriostatic and bactericidal properties, depending on the type of microorganism and the antibiotic concentration used. It is most effective against *Staphylococcus aureus* cocci, streptococcal group A, enterococci, and pneumococci. It inhibits the *Neisseriae* strain, and some strains of *Haemophilus influenzae*, *Pasteurellae multocidae*, *Brucellae*, *Rickettsiae*, and *Treponemae*. It is also effective against *Mycoplasma pneumoniae*, *Chlamydiae*, *Legionellae pneumophilae*, and some other atypical mycobacteria. The bioavailability of erythromycin is 30%–65%, and it is distributed in most tissues and body fluids. Plasma protein binding of erythromycin is 70%–90% and it is metabolized in the liver, partly with the formation of inactive metabolites with a t_1/2_ of about 1.4–2 h [42].

**Figure 2:**
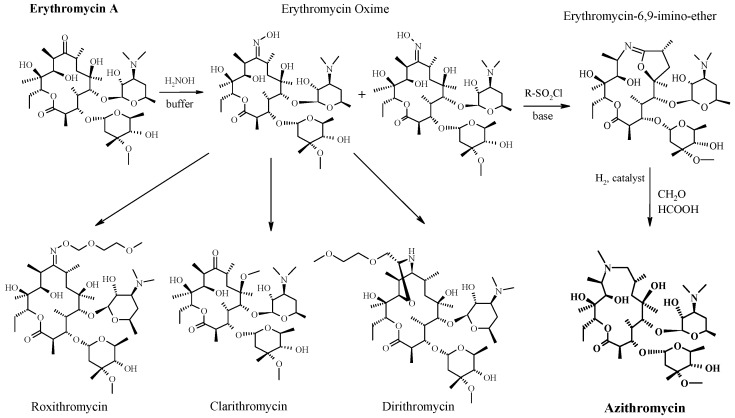
From Erythromycin A to other macrocyclic antibiotics (roxithromycin, dirithromycin, clarithromycin, etc.), including azithromycin, a “blockbuster” anti-infective drug [31].

Erythromycin, with its ten chiral centers and two sugar substituents (l-cladinose and d-desosamine, Figure 2), was a good starting point for numerous medicinal chemistry efforts for improvement of its biological profile (better activity, higher stability, and improved bioavailability) since the first generation of macrolides, which had low toxicity and good tolerability, were unstable in acidic media, had low toxicity and good tolerability. In the acidic environment of the stomach, erythromycin A is metabolized to its inactive 8,9-anhydroerythromycin-6,9-hemiketal and anhydroerythromycin-6,9:9,12-spiroketal. To improve the acidic stability and oral bioavailability of erythromycin A, the first generation of semisynthetic macrolides were prepared and introduced to medical practice. The main goal was to avoid ketal formation, initially by modifying the keto group or reactive hydroxyl. This approach resulted in various new chemical entities [43,44,45]. The most important erythromycin derivative from this group of new derivatives was erythromycin A oxime, prepared from erythromycin A and hydroxylamine hydrochloride in the presence of a weak base and buffer (Figure 2) [46,47,48]. The same was identified by chemists at PLIVA (the largest pharmaceutical company in Croatia and one of the leading companies in Southeast Europe and, today, a member of the Teva Group) and the work continued for the further synthesis of new, more active compounds, such as roxithromycin, dirithromycin, and clarithromycin. The roxithromycin enriched with an N-oxime side chain attached to the macrolactone ring [20], dirithromycin, in which the 9-keto group of the macrolactone ring was converted to an amino group [21], and clarithromycin, in which an additional methyl group in the 6-*O*-position, in comparison to erythromycin these modifications significantly improved acid-stability [22] (Figure 2).

The first 15-membered macrolide antibiotic on the market—azithromycin (9a-methyl-9-deoxo-9-dihydro-9a-aza-9a-homoerythromycin) (Figure 2), characterized by a basic nitrogen atom inserted into the macrocyclic ring, was synthesized in 1980 by a team of researchers at PLIVA Laboratories [49,50]. Twenty years later, in 2000, for their outstanding contribution to chemistry they received the medal of highest honor, “Heroes of Chemistry”, awarded by the American Chemical Society. The discovery of the 15-membered imino-ether, produced by Beckmann’s rearrangement of 9(E)-erythromycin A oxime to the amide, led to the production of a qualitatively new group of macrolide antibiotics, named the azalides [50,51,52,53]. The key reaction in the formation of azalides was established during the synthesis of O-sulfonyl derivatives of 9(E)-erythromycin A oxime [50,51]. Treatment of 9(E)-erythromycin A oxime with benzenesulfonyl chloride in an acetone-water mixture with sodium bicarbonate yielded an unexpected product, erythromycin-6,9-imino-ether (Figure 2) [51,52,53]. A breakthrough was the discovery of a way to open the smaller ring by hydration, and it was confirmed that a 15-membered macrocyclic ring with an “incorporated” amino group had been formed [51]. At that moment, the dogma that the macrocyclic erythromycin ring was responsible for antibiotic activity of macrolides was shattered. A new substance had similar activity against Gram-positive bacteria and significantly better activity against Gram-negative bacteria in comparison to erythromycin A [31]. The stability in an acid environment was improved, and acute toxicity decreased, but slightly less than erythromycin. However, this reaction was just one innovative step towards a much more important discovery. Reductive methylation of 9-dihydro-9-deoxo-9a-aza-9a-homoerythromycin A with formaldehyde and formic acid yielded a novel product, a significantly more potent and bioavailable macrolide antibiotic named azithromycin (Figure 2) [51,54]. Its broad spectrum of activity covered all relevant bacteria causing respiratory tract infections, including *Haemophilus influenzae* and *Moraxella catarrhalis*. Azithromycin was up to four times more potent than erythromycin against *Haemophilus influenzae* and *Neisseria gonorrhoeae*, and two-fold more potent against *Branhamella catarrhalis*, *Campylobacter* and *Legionella* sp., and significantly more potent in comparison to many genera of the family *Enterobacteriaceae*. Its minimal inhibitory concentration (MIC) for 90% of strains of *Escherichia*, *Salmonella*, *Shigella*, and *Yersinia* was ≤4 μg/mL, compared with 16 to 128 μg/mL for erythromycin [54]. Tests in animal model has shown that azithromycin treatment is able to clear chlamydial genital infection but is unable to eliminate chlamydial infection in the cecum within the same animal in doses which were effective in clearing the genital infection [55].

The in vivo tests showed that azithromycin is less than half as toxic as erythromycin (erythromycin i.v./p.o. LD50 is 360/4000 mg/kg, while azithromycin i.v./p.o. LD50 is 825/10,000 mg/kg) [52]. Furthermore, in all studies for in vivo efficacy against systemic infections in mice (by *S. aureus*, *S. typhimurium*, *S. pyogenes*, and *S. peneumoniae*), significant superiority of azithromycin was demonstrated, regardless of the administration route (subcutaneous or oral) [52,56]. Pharmacokinetic studies in mice, rats, rabbits, and dogs showed the unique properties of the new molecule, quite different than those of erythromycin. While serum levels of erythromycin oxime and amine, and levels of azithromycin were similar (several times higher than that of erythromycin), retention time was significantly longer, and urine levels were up to a hundred times higher. High and prolonged levels were observed in tissues of some organs, even 24 h after injection (liver, lung, intestine, and kidney). Brain tissue levels were low, indicating that it does not pass the brain-blood barrier [52,57,58]. Chronic toxicity was monitored in rats and dogs after 15 days and after one, three, and six months of daily dosing with 25, 50, 100, and 200 mg/kg azithromycin. Equal or small reversible adverse changes were observed with azithromycin than with erythromycin in animals given the same doses. Mutagenic, carcinogenic, and teratogenic tests were negative [52].

Clinical data exhibited close correspondence with the results obtained in animals. The serum elimination half-life was longer (41 h after oral administration, while for erythromycin it is 2 h), and renal excretion was prolonged, which indicated slow elimination from tissues. Plasma protein binding studies showed that 63% of azithromycin remained unbound in human serum; while in the case of erythromycin the percentage was only 24%, which is in line with the rapid tissue penetration of azithromycin, resulting in high tissue levels, prolonged tissue retention and high oral bioavailability (around 40%) [52]. The pharmacokinetic profile appears to be characterized by rapid and extensive uptake from the circulation into intracellular compartments. Azithromycin is subsequently slowly released, reflecting its long terminal phase elimination half-life relative to that of erythromycin [59]. These factors allowed for a single dose or single daily dose regimen in most infections, with the potential for increased compliance among outpatients for which a more frequent antimicrobial regimen might traditionally be indicated. Such a favorable pharmacokinetic profile was one of the key advantages of azithromycin, compared to the other antibacterials and macrolides [60], evaluated perform on upper respiratory tract infections (throat, sinus, and ear), lower respiratory tract infections (lung), sexually-transmitted diseases (urethritis, gonorrhea), and skin and subcutaneous tissue infections. The antibiotic was very well tolerated, and its antimicrobial spectrum, therapeutic efficacy and pharmacokinetic properties also suggested that a single daily dose of azithromycin could be effective in the treatment of all the above mentioned pathologies, as well as other infections caused by susceptible microorganisms [52,60].

Simultaneously with the testing for anti-infective properties, the physicochemical, pharmacological, and structural properties of azithromycin were investigated [61]. Azithromycin proved to be highly superior to that of erythromycin, relative to stability in acidic conditions [62]. With the determination of the crystal structure of the bacterial ribosome, various scientists tried to prepare binding complexes of ribosome and macrolide antibiotics. Analysis of the crystal structure of the large ribosomal subunit (50S) from *Deinococcus radiodurans*, complexed with azithromycin, showed that azithromycin exerts its antimicrobial activity by blocking the protein exit tunnel [26].

In 1988, azithromycin was introduced to the market by joint collaborative agreement between PLIVA (Sumamed^®^), and Pfizer (Zithromax^®^). After the year 2000, azithromycin became the market leader among antibiotics for respiratory tract infections. Pfizer’s Zithromax was one of the best-selling branded antibiotics in the United States and worldwide, with total sales peaking at US $2 billion in 2005 before starting to decline with the loss of patent protection in 2006 and the resulting generic competition. Azithromycin, one of the leading drugs of the late 20th century, represents an excellent example of a unique and rational medicinal chemistry and classical structure-activity relationship approach towards drug design. As a result, azithromycin is a drug discovery success story [31]. The discovery of azithromycin was not a typical example of a drug discovery project, since the drug was developed on the basis of its anti-infective activity, and its precise mechanism of action and interaction with the ribosome-RNA complex were only discovered later, subsequent to its successful marketing [63].

## 4. New Macrocyclic Ribosome Inhibitors as Possible Way to Avoid Resistance Problem

The microorganisms have developed several mechanisms to acquire resistance to antibiotics, including macrolides. One of these mechanisms responsible for the lower binding and deceasing of anti-infective activity of macrolides is the change in the structure of the ribosome target either by methylation or mutation of the 23S rRNA, or mutation and consequent structural changes in the L4 and L22 ribosomal proteins in 50S ribosomal subunit [64]. The resistance to macrolide antibiotics is due to modification of the ribosomal target by methylation or mutation, decreased uptake of the molecules, and active efflux of the drug.

A family of rRNA methyltransferases designated as the Erm enzymes (more than 30 proteins have been reported) in bacteria are responsible for the development of the bacterial resistance to the antibiotics due the methylation of the 23S rRNA, in particular at adenine 2058 [65]. Methylation at A2058 significantly influences the binding of macrolides to the ribosome target and it is responsible for development of a cross-resistance to macrolides [66]. The expression of *erm* genes of bacteria is inducible, regulated by silencing of gene expression and constitutive *erm* gene translation, or constitutive, and it has been reported that the ability of macrolides to induce the expression of the *erm* genes depends on their structure. Macrolides with a 14-member lactone ring are strong inducers of the *erm* genes and these include erythromycin [67]. In addition to the structural changes of the ribosome target in the resistance development, an important role in the resistance development is played by the efflux pumps. For example, the *msrA* gene encodes an ABC transporter in staphylococci and the *mefA* gene encodes the expression of a MFS pump in streptococci [68,69] which transport the antibiotics out of the bacteria.

Azithromycin exerts its antimicrobial activity by blocking the protein exit tunnel, but in contrast to other macrolides, this effect is possibly linked with the distinct binding sites, since an additional binding site has been also recognized within the large ribosomal subunit of *D. radiodurans* [26]. Nitrogen inserted into the lactone ring does not directly contribute to the binding of azithromycin to the ribosome, but this modification alters the conformation of the lactone ring sufficiently to induce novel contacts. One azithromycin molecule interacts with domains IV and V of 23S rRNA, whereas the second azithromycin interacts with two ribosomal proteins L4 and L22 and domain II of 23S rRNA [26], so azithromycin can be considered as a dual-binding ribosome inhibitor. Additionally, in an earlier example of binding of azithromycin on the *Escherichia coli* ribosome, it is shown that azithromycin binds in a two-step process—placing of the drug in a low-affinity site located in the upper part of the exit tunnel, and slow formation of a final complex that is much stronger and more potent in preventing the synthesis of the nascent peptide through the exit tunnel [24].

**Figure 3:**
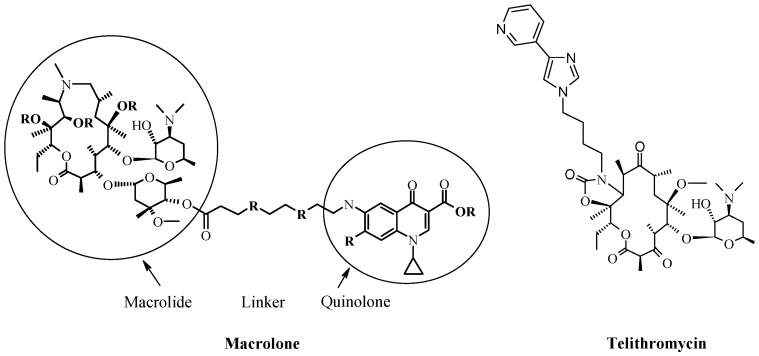
Schematic presentation of a macrolone molecule with its distinct moieties—macrolide, linker, and quinolone (**left**); and most common chemical modification and variation positions are marked with “R”. The structure of telithromycin, the first ketolide antibiotic clinically used to treat community-acquired pneumonia of mild to moderate severity (**right**).

In order to rationally design better drugs which would be able to overcome the existing resistance mechanisms, new directions in drug discovery have been evaluated, and rational approach to drug design, based on already-known mechanisms of drug actions has been applied. Multiple new macrolides were synthesized with the aim to inhibit the growth or to kill resistant bacterial strains. Concept of combining active macrolide scaffold and (hetero) aromatic unit via a flexible linker resulted with compounds showing remarkable antibacterial activity. This effect was first recognized in the ketolide group of macrolide antibiotics, where the cladinose sugar is substituted with a keto-group, with a cyclic carbamate group attached in the lactone ring, with telithromycin (Figure 3) [29,70,71,72,73]. These modifications enable ketolides to become more active on a much broader spectrum than other macrolides [70]. Furthermore, another potential proof of concept (at least in anti-bacterial activity) has been done on azitromycin 4″ derivatives (on cladinose sugar), where quinolone moieties have been linked with macrolactone ring, and activities toward strains which were not sensitive toward classical anti-infective macrolides have been significantly improved (Figure 3) [27].

A novel class of macrolide antibiotics—macrolones, which are derived from macrolides are comprised of a macrocyclic moiety, linker and either free or esterified quinolone 3-carboxylic group. Macrolones showed excellent antibacterial potency towards key erythromycin-resistant Gram-positive and Gram-negative bacterial strains. Compared to azithromycin or to quinolones, most of the compounds exhibited improved and superior in vitro potency against the key erythromycin-resistant respiratory pathogens [27,28,29]. They show activity on eryS- and MLSb-resistant *S. pneumoniae* (ribosome methylation as the major mechanisms of erythromycin resistance) in the murine pneumonia model [74]. For macrolones no demonstration has confirmed yet that their functional target is the ribosome or that they inhibit protein synthesis. However, due to flexibility of their structure, the macrolones are good candidates for possible multiple interactions with the ribosome, and the macrolide part of the macrolone should have a major role in this binding. As is the case with “classical” macrolides, which are known to possess favorable pharmacokinetic properties by accumulating in inflammatory cells, the macrolone class of compounds, with its distinct structural features, shows equal or better accumulation in inflammatory cells [75]. Unlike macrolides and ketolides, macrolones showed rapid bactericidal effects against *H. influenzae*, and they exhibited equal or lower in vitro resistance development potential than azithromycin and telithromycin in *S. pneumoniae*, *H. influenzae*, *S. aureus*, and *M. catarrhalis* [74]. Macrolones have a low clearance, large volume of distribution, and long half-life, complying with once-daily dosing potential [74]. However, even after considerable data about its in vitro activity, mode of action, in vivo efficacy, and recognizing macrolones as superior in comparison to the known macrolide antibiotics, true potential of that compound class should still be evaluated after obtaining more detailed structural insight into binding mode to the ribosome. Finally, a full pre-clinical package of ADME/Tox properties (in vitro/in vivo) of this, and similar, classes of conjugates of macrolides and other active moieties, such as quinolones, should be done before we can make a final conclusion about macrolones as a promising new class of compounds.

## 5. Concluding Remarks

The continuous increase of resistant bacterial strains causes a significant problem in modern health care and drug discovery. Discovery of first macrolide antibiotic erythromycin, and development of semisynthetic macrolides prepared and introduced to medical practice, such as roxithromycin, dirithromycin, spiramycin, clarithromycin, josamycin, and especially azithromycin, significantly changed anti-infective drug picture of 20th century. Azithromycin provided a considerable boost to the worldwide therapy of bacterial infections. Yet now, well into the 21st century, the ketolide group of antibiotics, such as telithromycin and solithromycin as its most important members, together with azithromycin and some of the other macrolide antibiotics, continue to reveal unexpected activities. These factors have maintained the intense research interest and may well spawn new derivatives with therapeutic effects well beyond the field of antibiotics. However, the microorganisms have developed several mechanisms to acquire resistance to all antibiotics, among them also to macrolides. Therefore, in order to design better drugs which would be able to overcome the existing resistance mechanisms, new rational directions in drug discovery and drug design have been and should be evaluated. In addition to the abovementioned ketolides, and azithromycin’s possible dual-binding mode of action, where one azithromycin molecule interacts with rRNA domains, and the second azithromycin interacts with two ribosomal proteins (in a two-step process), the concept of combining active macrolide scaffold and (hetero)aromatic unit via a flexible linker has yielded compounds showing remarkable antibacterial activity. The novel class of macrolide antibiotics—macrolones, which are derived from macrolides, and which comprise macrocyclic moiety, linker, and either free or esterified quinolone group, could be great examples of combining excellent antibacterial potency towards key erythromycin-resistant pathogens. The potential novel concept of dual-binding ribosome inhibition is a possible mechanism for preventing growing resistance development.

## Abbreviations

The following abbreviations are used in this manuscript:

rRNA	ribosomal Ribonucleic acid
tRNA	transfer Ribonucleic acid
MIC	Minimal inhibitory concentration
i.v	Intravenous injection, a route of administration of a drug
p.o	Per oral, a route of administration of a drug
LD50	The median lethal dose
EryS	Erythromycin susceptible
EryR	Erythromycin resistant
MLSb	Macrolide-Lincosamide-Streptogramin b

## References

[1] Labro M.T. (1998). Anti-inflammatory activity of macrolides: A new therapeutic potential?. J. Antimicrob. Chemother..

[2] Labro M.T. (2004). Macrolide antibiotics: Current and future uses. Expert Opin. Pharmacother..

[3] Čulić O., Eraković V., Parnham M.J. (2001). Anti-inflammatory effects of macrolide antibiotics. Eur. J. Pharmacol..

[4] Amsden G.W. (2005). Anti-inflammatory effects of macrolides—An underappreciated benefit in the treatment of community-acquired respiratory tract infections and chronic inflammatory pulmonary conditions?. J. Antimicrob. Chemother..

[5] Sassa K., Mizushima Y., Fujishita T., Oosaki R., Kobayashi M. (1999). Therapeutic effect of clarithromycin on a transplanted tumor in rats. J. Antimicrob. Chemother..

[6] Bukvić Krajačić M., Perić M., Smith K.S., Ivezić Schönfeld Z., Žiher D., Fajdetić A., Kujundžić N., Schönfeld W., Landek G., Padovan J. (2011). Synthesis, structure-activity relationship, and antimalarial activity of ureas and thioureas of 15-membered azalides. J. Med. Chem..

[7] Perić M., Fajdetić A., Rupčić R., Alihodžić S., Žiher D., Bukvić Krajačić M., Smith K.S., Ivezić-Schönfeld Z., Padovan J., Landek G. (2012). Antimalarial activity of 9a-*N* substituted 15-membered azalides with improved in vitro and in vivo activity over azithromycin. J. Med. Chem..

[8] Wolter J., Seeney S., Bell S., Bowler S., Masel P., McCormack J. (2002). Effect of long term treatment with azithromycin on disease parameters in cystic fibrosis: a randomised trial. Thorax.

[9] Marjanović N., Bosnar M., Michielin F., Willé D.R., Anić-Milić T., Čulić O., Popović-Grle S., Bogdan M., Parnham M.J., Eraković Haber V. (2011). Macrolide antibiotics broadly and distinctively inhibit cytokine and chemokine production by COPD sputum cells in vitro. Pharmacol. Res..

[10] Piacentini G.L., Peroni D.G., Bodini A., Pigozzi R., Costella S., Loiacono A., Boner A.L. (2007). Azithromycin reduces bronchial hyperresponsiveness and neutrophilic airway inflammation in asthmatic children: A preliminary report. Allergy Asthma Proc..

[11] Hernando-Sastre V. (2010). Macrolide antibiotics in the treatment of asthma. An update. Allergol. Immunopathol..

[12] Kino T., Hatanaka H., Hashimoto M., Nishiyama M., Goto T., Okuhara M., Kohsaka M., Aoki H., Imanaka H. (1987). FK-506, a novel immunosuppressant isolated from a *Streptomyces* I. Fermentation, isolation, and physico-chemical and biological characteristics. J. Antibiot..

[13] Vézina C., Kudelski A., Sehgal S.N. (1975). Rapamycin (AY-22,989), a new antifungal antibiotic. J. Antibiot..

[14] Vandeputte P., Ferrari S., Coste A.T. (2012). Antifungal resistance and new strategies to control fungal infections. Int. J. Microbiol..

[15] Omura S., Shiomi K. (2007). Discovery, chemistry, and chemical biology of microbial products. Pure Appl. Chem..

[16] Tacar O., Sriamornsak P., Dass C.R. (2013). Doxorubicin: An update on anticancer molecular action, toxicity and novel drug delivery systems. J. Pharm. Pharmacol..

[17] Hecht S.M. (2000). Bleomycin: New perspectives on the mechanism of action. J. Nat. Prod..

[18] Omura S. (2002). Macrolide Antibiotics. Chemistry, Biology and Practice.

[19] Kaufman H.E. (1961). Spiramycin. Arch. Ophthalmol..

[20] Puri S.K., Lassman H.B. (1987). Roxithromycin: A pharmacokinetic review of a macrolide. J. Antimicrob. Chemother..

[21] Fernandes P.B., Hardy D.J. (1988). Comparative in vitro potencies of nine new macrolides. Drugs Exp. Clin. Res..

[22] Watanabe Y., Morimoto S., Adachi T., Kashimura M., Asaka T. (1993). Selective methylation at the C-6 hydroxyl group of erythromycin A oxime derivatives and preparation of clarithromycin. J. Antibiot..

[23] Schlünzen F., Zarivach R., Harms J., Bashan A., Tocilj A., Albrecht R., Yonath A., Franceschi F. (2001). Structural basis for the interaction of antibiotics with the peptidyl transferase centre in eubacteria. Nature.

[24] Petropoulos A.D., Kouvela E.C., Starosta A.L., Wilson D.N., Dinos G.P. (2009). Time-resolved binding of azithromycin to *Excherichia coli* ribosomes. J. Mol. Biol..

[25] Petrov A.S., Bernier C.R., Hershkovits E., Xue Y., Waterbury C.C., Hsiao C., Stepanov V.G., Gaucher E.A., Grover M.A., Harvey S.C. (2013). Secondary structure and domain architecture of the 23S and 5S rRNAs. Nucleic Acids Res..

[26] Schlünzen F., Harms J.M., Franceschi F., Hansen A.S., Bartels H., Zarivach R., Yonath A. (2003). Structural basis for the antibiotic activity of ketolides and azalides. Structure.

[27] Fajdetić A., Cipcić Paljetak H., Lazarevski G., Hutinec A., Alihodzić S., Derek M., Stimac V., Andreotti D., Sunjić V., Berge J.M. (2010). 4″-*O*-(omega-Quinolylamino-alkylamino)propionyl derivatives of selected macrolides with the activity against the key erythromycin resistant respiratory pathogens. Bioorg. Med. Chem..

[28] Fajdetić A., Vinter A., Paljetak H.Č., Padovan J., Jakopović I.P., Kapić S., Alihodžić S., Filić D., Modrić M., Košutić-Hulita N. (2011). Synthesis, activity and pharmacokinetics of novel antibacterial 15-membered ring macrolones. Eur. J. Med. Chem..

[29] Kapić S., Cipčić Paljetak H., Palej Jakopović I., Fajdetić A., Ilijaš M., Stimac V., Brajša K., Holmes D.J., Berge J., Alihodžić S. (2011). Synthesis of macrolones with central piperazine ring in the linker and its influence on antibacterial activity. Bioorg. Med. Chem..

[30] Amsden G.W. (2001). Advanced-generation macrolides: Tissue-directed antibiotics. Int. J. Antimicrob. Agents.

[31] Jelić D., Mutak S., Lazarevski G. (2013). The azithromycin success story. Medicinal Chemistry in Drug Discovery. Design, Synthesis and Screening.

[32] Gaynor M., Mankin A.S. (2003). Macrolide antibiotics: Binding site, mechanism of action, resistance. Curr. Top. Med. Chem..

[33] Bulkley D., Innis C.A., Blaha G., Steitz T.A. (2010). Revisiting the structures of several antibiotics bound to the bacterial ribosome. Proc. Natl. Acad. Sci. USA.

[34] Cornish P.V., Ermolenko D.N., Noller H.F., Ha T. (2008). Spontaneous intersubunit rotation in single ribosomes. Mol. Cell.

[35] Ban N., Nissen P., Hansen J., Moore P.B., Steitz T.A. (2000). The complete atomic structure of the large ribosomal subunit at 2.4 A resolution. Science.

[36] Harms J., Schluenzen F., Zarivach R., Bashan A., Gat S., Agmon I., Bartels H., Franceschi F., Yonath A. (2001). High resolution structure of the large ribosomal subunit from a mesophilic eubacterium. Cell.

[37] Yonath A. (2005). Antibiotics targeting ribosomes: Resistance, selectivity, synergism, and cellular regulation. Annu. Rev. Biochem..

[38] Yu D., Zhang C., Qin P., Cornish V.P., Xu D. (2014). RNA-protein distance patterns in ribosomes reveal the mechanism of translational attenuation. Sci. China Life Sci..

[39] Rodnina M.V., Wintermayer W. (2003). Peptide bond formation on the ribosome structure and mechanism. Curr. Opin. Struct. Biol..

[40] Herman T. (2005). Drugs targeting the ribosome. Curr. Opin. Struct. Biol..

[41] Leclercq R., Courvalin P., Leclercq R., Rice L. (2010). Macrolides, lincosamides, and streptogramins. Antibiogram.

[42] Djokić S., Kobrehel G., Lopotar N., Kamenar B., Nagl A., Mrvos D. (1988). Erythromycin series. Part 13. Synthesis and structure elucidation of 10-dihydro-10-deoxo-11-methyl-11-azaerythromycin A. J. Chem. Res..

[43] Kirst H.A., Schoenfeld W., Kirst H.A. (2002). Macrolide Antibiotics.

[44] Neu H.C., Young L.S., Zinner S.H., Acar J.F., Dekker M. (1995). New Macrolides, Azalides and Streptogramins in Clinical Practice.

[45] Schoenfeld W., Mutak S., Schoenfeld W., Kirst H.A. (2002). Macrolide Antibiotics.

[46] Yang B.V., Goldsmith M., Rizzi A. (1994). A novel product from Beckmann rearrangement of erythromycin A 9(E)-oxime. Tetrahedron Lett..

[47] Fattori R., Pelacini F., Romagnano S., Fronza G., Rallo R. (1996). Unusual isoxazoline formation by intramolecular cyclization of (9*E*)-erythromycin A oxime. J. Antibiot..

[48] Djokić S., Tamburasev Z. (1967). 9-Amino-3-*O*-cladinosyl-6,11,12-trihydroxy 2,4,6,8,10,12-hexamethylpentadecane-13-olide. Tetrahedron Lett..

[49] Kobrehel G., Radobolja G., Tamburasev Z., Djokic S. (1982). 11-Aza-10-Deozo-10-Dihydroerythromycin A and Derivatives Thereof as Well as a Process for their Preparation. U.S. Patent.

[50] Kobrehel G., Djokic S. (1985). 11-Methyl-11-Aza-4-*O*-Cladinosyl-6-*O*-Desosamynyl-15-Ethyl-7,13,14-Trihydroxy-3,5,7,9,12,14-Hexamethyloxacyclopentadecane-2-One and Derivatives Thereof. U.S. Patent.

[51] Djokić S., Kobrehel G., Lazarevski G., Lopotar N., Tamburašev Z., Kamenar B., Nagl A., Vicković I. (1986). Ring expansion of erythromycin A oxime by the Beckmann rearrangement. J. Chem. Soc. Perkin Trans. I.

[52] Đokić S. (1988). From erythromycin to azithromycin—From macrolides to azalides. PLIVA Saopć..

[53] Mutak S. (2007). Azalides from azithromycin to new azalide derivatives. J. Antibiot..

[54] Retsema J., Girard A., Schelkly W., Manousos M., Anderson M., Bright G., Borovoy R., Brenan L., Mason R. (1987). Spectrum and mode of action of azithromycin (CP-62,993), a new 15-membered-ring macrolide with improved potency against gram-negative organisms. Antimicrob. Agents Chemother..

[55] Yeruva L., Melnyk S., Spencer N., Bowlin A., Rank R.G. (2013). Differential susceptibilities to azithromycin treatment of chlamydial infection in the gastrointestinal tract and cervix. Antimicrob. Agents Chemother..

[56] Bright G.M., Nagel A.A., Bordner J., Desai K.A., Dibrino J.N., Nowakowska J., Vincent L., Watrous R.M., Sciavolino F.C., English A.R. (1988). Synthesis, in vitro and in vivo activity of novel 9-deoxo-9a-aza-9a-homoerythromycin A derivatives; a new class of macrolide antibiotics, the azalides. J. Antibiot..

[57] Girard A.E., Girard D., English A.R., Gotz T.D., Cimochowski C.R., Faiella J.A., Haskell S.L., Retsema J.A. (1987). Pharmacokinetic and in vivo studies with azithromycin (CP-62,993), a new macrolide with an extended half-life and excellent tissue distribution. Antimicrob. Agent Chemother..

[58] Foulds G., Shepard R.M., Johnson R.B. (1990). The pharmacokinetics of azithromycin in human serum and tissues. J. Antimicrob. Chemother..

[59] Peters D.H., Friedel H.A., McTavish D. (1992). Azithromycin. A review of its antimicrobial activity, pharmacokinetic properties and clinical efficacy. Drugs.

[60] Schönwald S., Skerk V., Petricevic I., Car V., Majerus-Misic L., Gunjaca M. (1991). Comparison of three-day and five-day courses of azithromycin in the treatment of atypical pneumonia. Eur. J. Clin. Microbiol. Infect. Dis..

[61] Lazarevski G., Vinković M., Kobrehel G., Đokic S., Metelko B., Vikić-Topić D. (1993). Conformational analysis of azithromycin by nuclear magnetic resonance spectroscopy and molecular modelling. Tetrahedron.

[62] Fiese E.F., Steffen S.H. (1990). Comparison of the acid stability of azithromycin and erythromycin A. J. Antimicrob. Chemother..

[63] Hansen J.L., Ippolito J.A., Ban N., Nissen P., Moore P.B., Steitz T.A. (2002). The structures of four macrolide antibiotics bound to the large ribosomal subunit. Mol. Cell.

[64] O’Connor M., Gregory S.T., Dahlberg A.E. (2004). Multiple defects in translation associated with altered ribosomal protein L4. Nucleic Acids Res..

[65] Bailey M., Chettiath T., Mankin A.S. (2008). Induction of *erm*(C) expression by noninducing antibiotics. Antimicrob. Agents Chemother..

[66] Maravić G. (2004). Macrolide resistance based on the Erm-mediated rRNA methylation. Curr. Drug Targets Infect. Disord..

[67] Leclercq R., Courvalin P. (2002). Resistance to macrolides and related antibiotics in *Streptococcus pneumonia*. Antimicrob. Agents Chemother..

[68] Cai Y., Kong F., Gilbert G.L. (2007). Three new macrolide efflux (*mef*) gene variants in *Streptococcus agalactiae*. J. Clin. Microbiol..

[69] Burnie J.P., Matthews R.C., Carter T., Beaulieu E., Donohoe M., Chapman C., Williamson P., Hodgetts S.J. (2000). Identification of an immunodominant ABC transporter in methicillin-resistant *Staphylococcus aureus* infections. Infect. Immun..

[70] Scheinfeld N. (2004). Telithromycin: A brief review of a new ketolide antibiotic. J. Drugs Dermatol..

[71] Agouridas C., Denis A., Auger J.M., Benedetti Y., Bonnefoy A., Bretin F., Chantot J.F., Dussarat A., Fromentin C., D’Ambrières S.G. (1998). Synthesis and antibacterial activity of ketolides (6-*O*-methyl-3-oxoerythromycin derivatives): A new class of antibacterials highly potent against macrolide-resistant and -susceptible respiratory pathogens. J. Med. Chem..

[72] Evrard-Tedeschi N., Gharbi-Benarous J., Gaillet C., Verdier L., Bertho G., Lange C., Parent A., Girault J.-P. (2000). Conformations in solution and bound to bacterial ribosomes of ketolides, HMR 3647 (telithromycin) and RU 72366: A new class of highly potent antibacterials. Bioorg. Med. Chem..

[73] Bukvić Krajačić M., Novak P., Cindrić M., Brajša K., Dumić M., Kujundžić N. (2007). Azithromycin-sulfonamide conjugates as inhibitors of resistant *Streptococcus pyogenes* strains. Eur. J. Med. Chem..

[74] Cipcic Paljetak H., Banjanac M., Ergovic G, Peric M., Padovan J., Dominis-Kramaric M., Kelneric Z., Verbanac D., Holmes D.J., Erakovic Haber V. Macrolones—Novel class of macrolide antibiotics active against key resistant respiratory pathogens. Proceedings of the 53rd ICAAC Meeting.

[75] Munić Kos V., Koštrun S., Fajdetić A., Bosnar M., Kelnerić Ž., Stepanić V., Eraković Haber V. (2013). Structure-property relationship for cellular accumulation of macrolones in human polymorphonuclear leukocytes (PMNs). Eur. J. Pharm. Sci..

